# Supplementary material for: The Habitual Diet of Dutch Adult Patients with Eosinophilic Esophagitis Has Pro-Inflammatory Properties and Low Diet Quality Scores
Source: Nutrients. 2021 Jan 13;13(1):214. doi: 10.3390/nu13010214 (PMC7828600; doi:10.3390/nu13010214)
Supplement: Supplementary file 1 [file nutrients-13-00214-s001.pdf]

**Supplementary Table S1.** Nutrients calculated on the basis of 3-day food diaries and distribution of food groups used.

**Energy and nutrients calculated (*n* = 32):**

Energy (kcal), protein (g and energy percentage), fat (g and energy percentage), saturated fat (g and energy percentage), carbohydrates (g and energy percentage), linoleic acid (g), $\alpha$-linolenic acid (ALA), eicosapentaenoic acid (EPA) and docosahexaenoic acid (DHA) (g), dietary fiber (g), alcohol (g), calcium (mg), iron (mg), sodium (mg), potassium (mg), magnesium (mcg), zinc (mg), selenium (mcg), iodine (mg), copper (mg), phosphorus (mg), retinol activity equivalent (RAE = retinol + precursor carotenoids) (mcg), vitamins A (mcg), B1 (mg), B2 (mg), B3 (mg), B6 (mg), folic acid (in mcg; folate in mcg and folate equivalents), B12 (mcg), C (mg), D (mg) and E (mg).

**Distribution of food groups calculated (*n* = 38):**

Ingested number of potatoes and other turnips in grams/day
Ingested number of vegetables in grams/day
Ingested number of legumes in grams/day
Ingested amount of fruit, nuts and olives in grams/day
Ingested amount of fruit in grams/day
Ingested number of dairy products in grams/day
Ingested amount of milk in grams/day
Ingested amount of skimmed milk in grams/day
Ingested amount of semi-skimmed/whole milk in grams/day
Ingested amount of yogurt in grams/day
Ingested amount of low-fat yogurt in grams/day
Ingested amount of semi-fat/full-fat yogurt in grams/day
Ingested amount of cheese in grams/day
Ingested amount of breakfast cereals and other grain products in grams/day
Ingested amount of bread in grams/day
Ingested amount of white bread in grams/day
Ingested amount of multigrain/whole wheat bread in grams/day
Ingested amounts of wheat bread in grams/day
Ingested amount of breakfast cereals in grams/day
Ingested amount of pasta and rice in grams/day
Ingested amount of meat and meat products in grams/day
Ingested amount of fish and seafood in grams/day
Ingested amount of egg and egg products in grams/day
Ingested amount of added fat (margarine, butter, oil, cooking fat) in grams/day
Ingested amount of low-fat margarine and margarine in grams/day
Ingested amount of butter in grams/day
Ingested amount of vegetable oil in grams/day
Ingested amount of olive oil in grams/day
Ingested number of other oils than olive oil in grams/day
Ingested amount of baking and roasting products in grams/day

| |
|---|
| Ingested amount of sugar and sweets in grams/day |
| Ingested number of pastries, cakes and biscuits in grams/day |
| Ingested number of non-alcoholic beverages in ml/day |
| Ingested number of alcoholic beverages in ml/day |
| Ingested number of spices and sauces in grams/day |
| Ingested amount of soup and broths in grams/day |
| Ingested number of other products in grams/day |
| Ingested amount of soy products in grams/day |

Distribution of food groups is based on the Dutch National Food Consumption Survey (DNFCS) [18].

## Supplementary File S1 – Calculation and validation of the Diet Quality Indices (DQI)

### Materials and Methods

*PANDiet*

The PANDiet is a nutrient-based DQI which measures the adequacy of intake of 25 macro- and micronutrients in comparison to nutritional recommendations: protein, total fat, saturated fatty acids (SFA), linoleic acid (LA), $\alpha$-linolenic acid (ALA), the sum of eicosapentaenoic acid (EPA) and docosahexaenoic acid (DHA), total carbohydrate, dietary fiber, vitamins A, B1, B2, B6, B12, C, D, E, calcium, copper, iodine, iron, magnesium, potassium, selenium, sodium, and zinc (Supplementary Table S2) [1].

It is based on two sub-scores: the Adequacy sub-score and the Moderation sub-score, and relies on the calculation of the probability of adequacy for each available nutrient, ranging from 0 to 1, where 1 represents a 100% probability that the intake is adequate according to a reference value. The calculation of the probability takes into account the number of days of dietary assessment, the mean intake and day-to-day variability in intake, the nutrient reference value and inter-individual variability. The adequate intake of a nutrient was assumed to be the level likely to satisfy the nutrient requirement and unlikely to be excessive. Consequently, the Adequacy sub-score computed the probability of adequacy for nutrients for which the usual intake should be above a reference value (the higher the sub-score, the better the intake satisfies the nutrient requirement). The Moderation sub-score computed the probability of adequacy for nutrients for which the usual intake should not exceed a reference value (the higher the sub-score, the less likely the intake is excessive). All of the probabilities of adequacy within each sub-score were equally weighted. The PANDiet score is taken as the mean of the Adequacy and Moderation sub-score and ranges from 0–100; the higher the score, the better the nutrient adequacy of the diet [1].

**Supplementary Table S2.** Estimated average requirements used in the calculation of the PANDiet.

| | Men 19-30 years | Women 19-30 years | Men 31-50 years | Women 31-50 years | Men 51-69 years | Women 51-69 years | Variability |
|---|---|---|---|---|---|---|---|
| **Adequacy sub-score** | | | | | | | |
| Protein | 6 en% | 7 en% | 6 en% | 7 en% | 7 en% | 8 en% | 12.5 % |
| Total Fat | 20 en% | 20 en% | 20 en% | 20 en% | 20 en% | 20 en% | 0 % |
| LA[1] (C18:2 n-6) | 2 en% | 2 en% | 2 en% | 2 en% | 2 en% | 2 en% | 15 % |
| ALA[1] (C18:3 n-3) | 1 en% | 1 en% | 1 en% | 1 en% | 1 en% | 1 en% | 15 % |
| EPA[1] + DHA[1] | 200 mg | 200 mg | 200 mg | 200 mg | 200 mg | 200 mg | 15 % |

| | | | | | | | |
|---|---|---|---|---|---|---|---|
| Total carbohydrate | 40 en% | 40 en% | 40 en% | 40 en% | 40 en% | 40 en% | 0 % |
| Dietary fiber | 3.4 g/MJ | 3.4 g/MJ | 3.4 g/MJ | 3.4 g/MJ | 3.4 g/MJ | 3.4 g/MJ | 15 % |
| Vitamin A | 615 µg RE | 525 µg RE | 615 µg RE | 525 µg RE | 615 µg RE | 525 µg RE | 15% |
| Thiamin (Vitamin B1) | 0.072 mg/MJ | 0.072 mg/MJ | 0.072 mg/MJ | 0.072 mg/MJ | 0.072 mg/MJ | 0.072 mg/MJ | 15% |
| Riboflavin (Vitamin B2) | 1.3 mg | 1.3 mg | 1.3 mg | 1.3 mg | 1.3 mg | 1.3 mg | 15 % |
| Vitamin B6 | 1.1 mg | 1.1 mg | 1.1 mg | 1.1 mg | 1.3 mg | 1.1 mg | 20 % |
| Vitamin B12 | 2.0 µg | 2.0 µg | 2.0 µg | 2.0 µg | 2.0 µg | 2.0 µg | 20 % |
| Vitamin C | 60 mg | 50 mg | 60 mg | 50 mg | 60 mg | 50 mg | 25 % |
| Vitamin D | 10 µg[2] | 10 µg[2] | 10 µg[2] | 10 µg[2] | 10 µg[2] | 10 µg[2] | 15 % |
| Vitamin E | 13 mg[2] | 11 mg[2] | 13 mg[2] | 11 mg[2] | 13 mg[2] | 11 mg[2] | 15 % |
| Calcium | 860 mg[3] | 860 mg[3] | 750 mg[4] | 750 mg[4] | 750 mg | 1100 mg[2] | 15 % |
| Copper | 0.7 mg | 0.7 mg | 0.7 mg | 0.7 mg | 0.7 mg | 0.7 mg | 15 % |
| Iodine | 150 µg[2] | 150µg[2] | 150 µg[2] | 150 µg[2] | 150 µg[2] | 150 µg[2] | 20 % |
| Iron | 6 mg | 7 mg | 6 mg | 7 mg[5] | 6 mg | 6 mg[6] | 20 % |
| Magnesium | 350 mg[2] | 300 mg[2] | 350 mg[2] | 300 mg[2] | 350 mg[2] | 300 mg[2] | 10 % |
| Potassium | 3.5 g[2] | 3.5 g[2] | 3.5 g[2] | 3.5 g[2] | 3.5 g[2] | 3.5 g[2] | 15 % |
| Selenium | 70 µg[2] | 70 µg[2] | 70 µg[2] | 70 µg[2] | 70 µg[2] | 70 µg[2] | 15 % |
| Zinc | 6.4 mg | 5.7 mg | 6.4 mg | 5.7 mg | 6.4 mg | 5.7 mg | 15 % |
| **Moderation sub-score** | | | | | | | |
| Protein | 25 en% | 25 en% | 25 en% | 25 en% | 25 en% | 25 en% | 0 % |
| Total carbohydrate | 60 en% | 60 en% | 60 en% | 60 en% | 60 en% | 60 en% | 0 % |

| | | | | | | | |
|---|---|---|---|---|---|---|---|
| Total fat | 40 en% | 40 en% | 40 en% | 40 en% | 40 en% | 40 en% | 0 % |
| Saturated Fatty Acids | 10 en% | 10 en% | 10 en% | 10 en% | 10 en% | 10 en% | 15 % |
| Sodium | 2.4 g | 2.4 g | 2.4 g | 2.4 g | 2.4 g | 2.4 g | 15 % |
| *Tolerable Upper Intakes Limits for potential penalties*[7] | | | | | | | |
| Retinol (Vitamin A) | 3000 µg (women 51-69: 1500 µg) | | | | | | |
| Vitamin B6 | 25 mg | | | | | | |
| Vitamin D | 100 µg | | | | | | |
| Vitamin E | 300 mg | | | | | | |
| Calcium | 2500 mg | | | | | | |
| Copper | 5 mg | | | | | | |
| Iodine | 600 µg | | | | | | |
| Iron | 60 mg | | | | | | |
| Selenium | 300 µg | | | | | | |
| Zinc | 25 mg | | | | | | |

[1]Linoleic acid (LA); $\alpha$-linolenic acid (ALA), Eicosapentaenoic acid (EPA), Docosahexaenoic acid (DHA); [2]Adequate intake instead of estimated average requirement; [3]Estimated average requirement refers to 18-24 year olds; [4]Estimated average requirement refers to 25-50 year olds; [5]Estimated average requirements refers to premenopausal women; [6]Estimated average requirements refers to postmenopausal women; [7]Based on recommendations from the Scientific Committee on Food [2]. The recommendations were based on the recommendations of the Dutch Health Council [3, 4]

**References**


[1] Bianchi CM, Mariotti F, Verger EO, Huneau J-F. Pregnancy Requires Major Changes in the Quality of the Diet for Nutritional Adequacy: Simulations in the French and the United States Populations. PLOS ONE. 2016;11:e0149858.

[2] Scientific Committee on Food Scientific Panel on Dietetic Products Nutrition and Allergies. Tolerable Upper Intake Levels for Vitamins and Minerals 2006.

[3] Gezondheidsraad. Voedingsnormen voor vitamines en mineralen voor volwassenen. 2018.

[4] Gezondheidsraad. Voedingsnormen: energie, eiwitten, vetten en verteerbare koolhydraten. 2001.
